# Effect of defects on reaction of NiO surface with Pb-contained solution

**DOI:** 10.1038/srep44805

**Published:** 2017-03-20

**Authors:** Jongjin Kim, Binyang Hou, Changyong Park, Chi Bum Bahn, Jason Hoffman, Jennifer Black, Anand Bhattacharya, Nina Balke, Hawoong Hong, Ji Hyun Kim, Seungbum Hong

**Affiliations:** 1 Materials Science Division, Argonne National Laboratory, Lemont, IL 60439, USA; 2 HPCAT, Geophysical Laboratory, Carnegie Institution of Washington, Argonne, IL 60439, USA; 3 School of Mechanical Engineering, Pusan National University, Busan 46241, Republic of Korea; 4 Center for Nanophase Materials Sciences, Oak Ridge National Laboratory, Oak Ridge, TN 37831, USA; 5 Advanced Photon Source, Argonne National Laboratory, Lemont, IL 60439, USA; 6 Department of Nuclear Engineering, School of Mechanical and Nuclear Engineering, Ulsan National Institute of Science and Technology (UNIST), Ulsan 44919, Republic of Korea; 7 Department of Materials Science and Engineering, KAIST, Daejeon 34141, Republic of Korea

## Abstract

In order to understand the role of defects in chemical reactions, we used two types of samples, which are molecular beam epitaxy (MBE) grown NiO(001) film on Mg(001) substrate as the defect free NiO prototype and NiO grown on Ni(110) single crystal as the one with defects. *In-situ* observations for oxide-liquid interfacial structure and surface morphology were performed for both samples in water and Pb-contained solution using high-resolution X-ray reflectivity and atomic force microscopy. For the MBE grown NiO, no significant changes were detected in the high-resolution X-ray reflectivity data with monotonic increase in roughness. Meanwhile, in the case of native grown NiO on Ni(110), significant changes in both the morphology and atomistic structure at the interface were observed when immersed in water and Pb-contained solution. Our results provide simple and direct experimental evidence of the role of the defects in chemical reaction of oxide surfaces with both water and Pb-contained solution.

Physical and electrical properties of ionic crystals are dominated by the structure and concentration of the defects in many cases^1^,^2^,^3^,^4^. Furthermore, the defects are often responsible for mass transport, which determine materials property for advanced functionalities such as diffusion barriers^5^ and oxygen ion conductors^6^. For oxidation/corrosion on the metals, the defects (e.g., vacancies, interstitial atoms, dislocations, grain boundaries) accelerate the reaction process by providing a pathway for fast oxygen diffusion or a favorable place for the reaction to occur^7^. When metals react with oxygen, they form a solid oxide film or scale on the surface under most conditions. Depending on the oxidation conditions (e.g., temperature, time, pressure, gas composition), the oxide films may have varying microstructures and morphologies, and usually develop microcracks or microchannels^8^,^9^.

Many studies were performed to investigate the formation and accumulation of defects on oxide and its effect on the oxidation/corrosion^10^,^11^. Localized corrosion such as pitting corrosion has been of particular interest as it is one of the major degradation mechanisms affecting the integrity of materials, which occurs around defects^12^,^13^. Meletis and Lian^14^ proposed the vacancy/dislocation interaction mechanism of transgranular stress corrosion cracking as the subsurface vacancies interact with dislocations promoting their motion and modifying their configurations. Burstein *et al*.^15^ reported a mechanism of pitting corrosion, and demonstrated that pit nucleation occurs at preferential sites for some metals. Some impurities such as Pb, Cl and S accelerate the oxidation and corrosion, and although there are a lot of studies on investigation of the corrosion behavior in such impurities^16^,^17^,^18^,^19^,^20^ the interaction between impurities and defects remains still elusive if not fully understood.

In case of the nuclear industry, corrosion and cracking of structural materials in nuclear power plants is one of the major issues for plant safety and life extension. In pressurized water reactors (PWRs), a steam generator (SG) consists of thousands of metal tubings made of nickel-base alloys, which suffer from corrosion and stress corrosion cracking (SCC)^21^,^22^. Recent laboratory test data indicated that Alloy 690 used for tubing could be susceptible to corrosion and SCC under lead (Pb)-contaminated water and even more susceptible than Alloy 600 especially in high pH solution^23^. To understand the mechanism, the process of the interaction between Pb contained solution and surface oxide (hydroxide) needs to be observed from the very beginning.

At this point, we raise a fundamental question of “Can we design a model experiment to investigate the role of defects in the chemical reaction between the oxide surface and Pb contained solution? ” We used molecular beam epitaxy (MBE)^24^,^25^,^26^ as a tool to create defect-free NiO thin films on Mg(001) substrate, which can grow high-purity single crystal quality epitaxial films via operating in ultra-high vacuum (UHV) chamber and using high purities of the beam fluxes in the Center for Nanoscale Materials at Argonne National Laboratory. For the oxide films with defects, we used the process of growing polycrystalline NiO thin films on Ni(110) single crystals in UHV chamber reported elsewhere^27^,^28^. Using the samples with and without defects, we directly investigated the role of defects in Ni oxide thin films on their chemical reaction in water and Pb-contained solution. High resolution X-ray reflectivity was adopted to measure the interface structure between oxide and water or oxide and Pb-contained solution, and *in-situ* atomic force microscopy were used to investigate the morphology changes of oxide films in water and Pb-contained solution.

## Results and Discussion

In order to explore the effect of defects in NiO layer on the chemical reaction between NiO layer and water as well as Pb contained solution, we prepared two types of NiO: one is as-grown NiO on Ni(110) substrates where we expect to have defects such as phase boundary, and the other is MBE grown epitaxial NiO on MgO(001), which is relatively free from such (defect-free NiO). The changes in interfacial structure and surface morphology in water and Pb-contained solution were investigated by high resolution X-ray reflectivity (HRXR) and *in-situ* atomic force microscopy (AFM).

**Figure 1:**
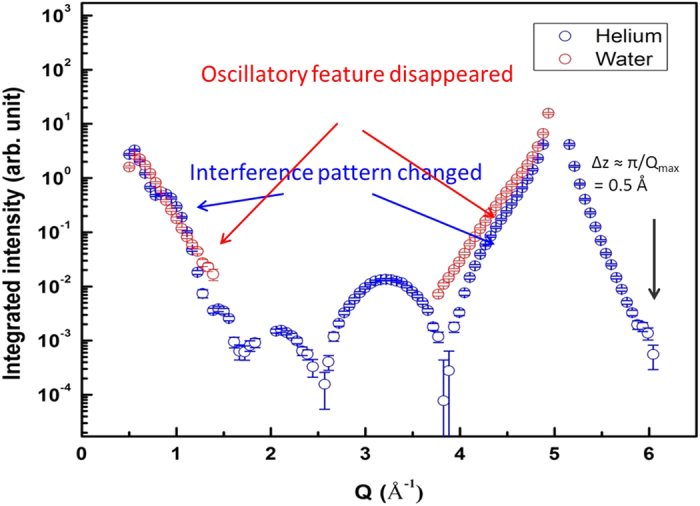
X-ray reflectivity of as grown NiO on Ni(110) single crystal exposed to helium and water environments.

Figure 1 shows the measured X-ray reflectivity data from the as-grown NiO on single crystal Ni(110) surface in helium environment and in deionized water, respectively. For the measured reflectivity in helium environment, the reflectivity shows distinct beating patterns at lower *q* side corresponding to the existence of at least two layers with different densities. However, near the substrate (220) Bragg peak, the regular film fringes appeared along the substrate reflectivity. This is a strong evidence that one of these layers have epitaxial relationship with the substrate, which suggests the existence of a crystalline NiO layer. As the film Bragg peak shows only one epitaxial layer without complicated beating pattern, we think that the other layer is either amorphous or polycrystalline phase^29^.

On the other hand, the reflectivity measured in deionized water from the identical surface shows completely different features in the intensity distribution compared to that measured in helium environment. First, the intensities at the mid-zone and higher momentum transfers (q) above the substrate (220) Bragg peak were hardly measurable while asymmetric intensity distribution before and after the Bragg peak were accompanied, which is similar to the effects of surface defects or defect clusters on the X-ray reflectivity intensities^30^. Second, the distinct film fringes with two layers and the epitaxial interference of the film Bragg peak disappeared completely. Two possibilities are suggested for these observations: one is that the as-grown NiO layer, regardless of its phase, interacts with water to be dissolved and the metal surface starts to form nickel hydroxide ad-layers whereas the other is that the intense X-ray generates reactive radicals in water nearby the oxide surface and causes secondary beam damage effects (e.g., continuous etching of the bare surface as well as oxide dissolution)^31^,^32^. We conducted an experiment to explore the X-ray beam damage effect, which will be discussed in the latter part of our discussion. It should be noted that we were not able to measure the X-ray reflectivity in Pb contained solution due to the disappearance of surface crystallinity when the sample was in contact with water.

**Figure 2:**
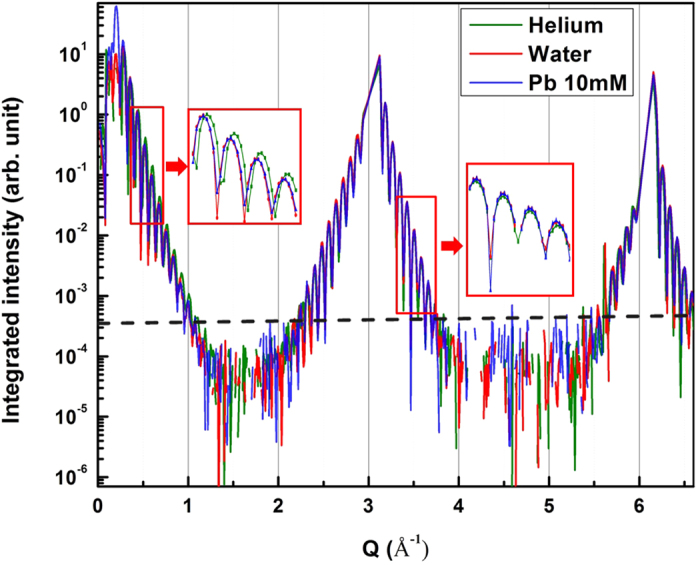
X-ray reflectivity of MBE grown NiO on MgO(001): in helium gas, and immersed in deionized water, and in 10 mM Pb-contained solution.

Figure 2 shows the X-ray reflectivity results of MBE grown nickel oxide on MgO substrate in helium, deionized water and 10 mM Pb-contained solution, respectively. The red boxes show the magnified images of the reflectivity close to the right shoulder of each Bragg peak. Commonly for all three cases, at the MgO(002) substrate Bragg peak, the film fringes at the left and right sides of the Bragg peak show slightly different features, of which difference is more prominent at MgO(004) Bragg peak. It results from d-spacing difference between MgO substrate and NiO films. And, at low q region, there is a phase shift and intensity reduction in the reflectivity of solution environment (deionized water or 10 mM Pb contained solution), due to the existence of liquid phase on the solid surface, relative to that of helium environment. As the modulus of liquid structure factor rapidly decreases with q^33^ the effect of existence of water phase on the observed intensity distribution is appearing only within low q range. Whether the ordering of liquid phases on the solid film is “layered” or follows “error-function” is undistinguishable, however, due to the overwhelming film fringe signals. The difference in the reflectivity data between de-ionized water and 10 mM Pb-contained solution in their intensity distribution is even smaller so that they are hardly distinguishable. Although the experiment could be better tailored to possibly separate out the critical difference between two liquid phases, we interpret the result as the MBE-grown nickel oxide is relatively inert both in pure water and in Pb-contained solution at room temperature compared to that grown on Ni(110) substrate.

**Figure 3:**
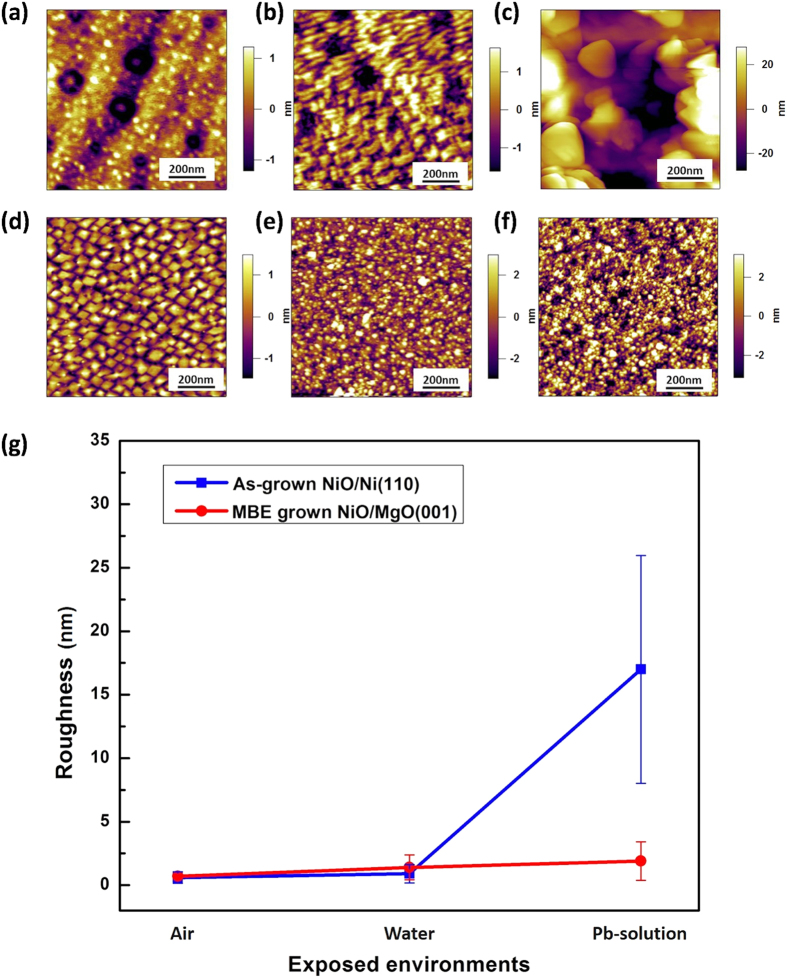
*In-situ* AFM images for as-grown NiO on Ni(110) (**a**–**c**) and MBE grown NiO on MgO(001) (**d**–**f**) in air (**a**), (**d**), water (**b**), (**e**) and 10 mM Pb-contained solution (**c**), (**f**) for 15 hrs, respectively. (**g**) Plot of roughness in each environment.

Figure 3 shows the *in-situ* AFM images for as-grown NiO on Ni(110) and MBE grown NiO on MgO(001) in air, water and 10 mM Pb-contained solution for 15 hrs. For the MBE grown NiO, the surface morphology shows dense diamond shaped facets with root-mean-square (rms) roughness of 0.7 nm followed by round shapes in water and Pb-contained solution with monotonous increase in rms roughness to 1.4 nm and 1.9 nm, respectively. For as-grown NiO on Ni(110) substrate, in contrast, the morphology in air shows a flat surface with rms roughness of ≈0.60 nm. Large and small crater-shaped spots are randomly distributed on the surface. After exposure in water for 15 hrs, the surface morphology changed to square shape due to the surface reconstruction by oxygen in water. The rms roughness slightly increased to ≈0.88 nm. In 10 mM Pb-contained solution, the surface was covered by crystallite particles with 0.1~0.3 μm size with drastic increase in rms roughness up to ≈17 nm. As evident from Fig. 3(c), the surface morphology underwent a significant change in Pb-contained solution. Figure 3(g) shows the roughness changes in air, water and 10 mM Pb-contained solution for both samples. For the MBE grown NiO, the roughness shows monotonous increase with change in environment from air to Pb-contained solution, while, in the case of as-grown NiO, the roughness shows drastic increase between water and Pb contained solution.

**Figure 4:**
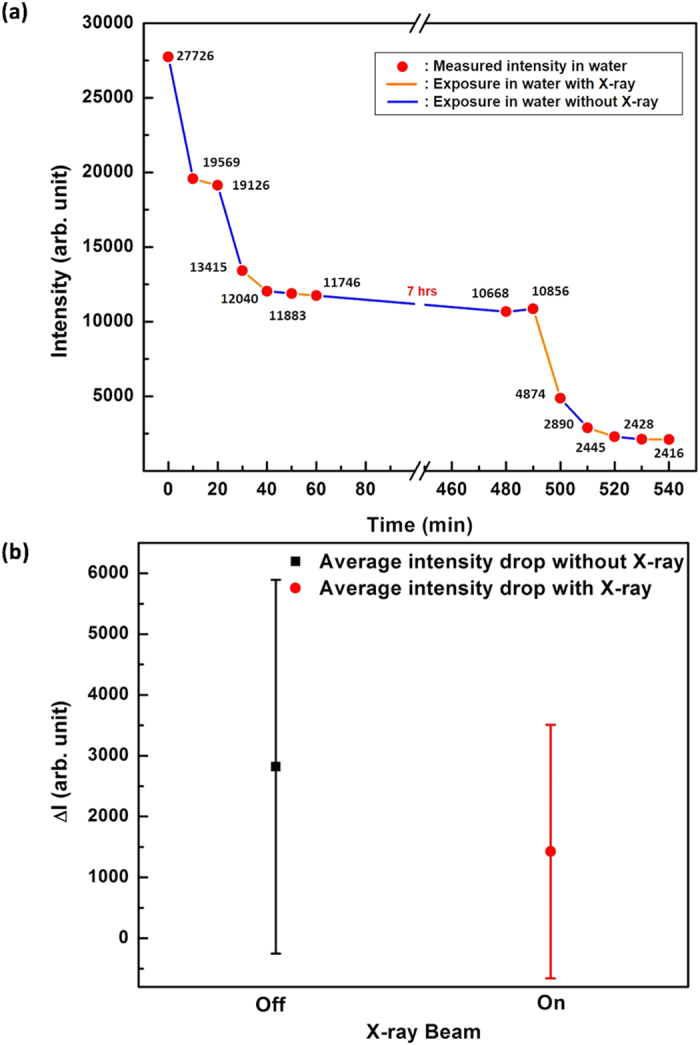
(**a**) Measured reflectivity intensity of NiO/Ni(110) with/without X-ray in water as function of time at L = 0.25 r.l.u. (near the Bragg peak). (**b**) Plot of average intensity drop with and without X-ray exposure.

In order to investigate the role of X-ray irradiation in the drastic decrease of the X-ray reflectivity intensity in water for as-grown NiO, we designed and performed continuous measurement of X-ray reflectivity of NiO immersed in water near the Bragg peak (L = 0.25 r.l.u.) with and without X-ray irradiation for a given period of time in an alternating manner as shown in Fig. 4(a). The red circles represent the measured intensity at each time, and the orange and blue lines represent the exposure in water with and without X-ray irradiation, respectively. Within 1 hr of immersion in water, the intensity decreases as a function of time even without X-ray irradiation and saturates after 60 mins. After 7 hrs immersion without X-ray, no significant intensity drop was observed before irradiation, while after exposure to X-ray, the intensity drastically started to drop and reached at a new saturation value. Within additional 1 hr, the intensity drop was caused probably by the chemical interaction between oxide and water until a certain saturation point. After saturation, the intensity was maintained whereas, after irradiation for 10 mins, the intensity started to drop and reached a new saturation level.

At a glance on Fig. 4, it seems rather difficult to conclude whether X-ray takes a role for the chemical reaction or not. Before further analyzing our data, it is worth mentioning relevant research reports on the role of X-ray irradiation on the chemical reaction of the oxide layer with water. Synchrotron based X-ray beams can affect the charge, bond and orbital states of strongly correlated systems^34^,^35^,^36^,^37^. Furthermore, ionization of molecules by X-ray can lead to radiolysis and formation of highly reactive free radicals. These radicals may then react chemically with neighbouring materials even after the original radiation has stopped. However, most studies performed to explore the effect of ionizing radiation and corrosion in nuclear materials was related to the 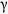-radiation, which has higher energy than X-ray. For example, Daub *et al*.^38^ revealed that the rate of carbon steel corrosion depends on the concentration of H_2_O_2_ which results from water radiolysis, and OH^−^ accelerates the corrosion process. Water radiolysis is the decomposition of water molecules due to the ionizing radiation, which creates ^•^HO radical, H^•^ atom, HO_2_^•^, H_3_O^+^, OH^−^, H_2_O_2_ and H_2_^39^. However, the X-ray energy that we used for our study is 17 keV, which is much lower than that used (~GeV) for the water radiolysis study. From this reasoning, we presumed that the beam damage of X-ray might be negligible.

To validate our presumption of negligible X-ray beam damage effect, we reorganized Fig. 4(a) so that we could compare the difference between the X-ray reflectivity intensities with and without X-ray exposure as shown in Fig. 4(b). Figure 4(b) shows the average reflectivity plot with and without X-ray exposure. We performed two-sample t-test to determine whether two samples are likely to have come from same two underlying populations that have the same mean. The p-value of our t-test is 0.635, much larger than the threshold value of 0.05, indicating that there is no significant statistical difference between the mean values of X-ray reflectivity with and without X-ray exposure. Furthermore, the X-ray reflectivity for the MBE grown NiO sample remained constant for more than a day. Therefore, we confirmed that X-ray did not affect the chemical reaction on the surface in our system.

As such, we can conclude that the different behaviors observed from two model systems, i.e., defect-free and defect-rich Ni oxide layers stem from the existence of the defects and neither from the bulk part of the oxide layer nor the external x-ray interacting with the water molecules. Indeed, the effect of defects on the chemical reaction between oxide film and water molecules has been reported by previous studies. Barbier *et al*.^40^ suggested that the chemical reaction of NiO(111) in water mainly takes place at defects, and Kitakatsu *et al*.^41^ claimed that the Ni(100) areas do not adsorb hydroxyl groups on regular sites but possibly on defect sites. Kofstad^42^ reported a comprehensive study on the importance of lattice, grain boundary and dislocation for metal oxide, but its defect structure and transport properties are still subject to considerable discussion.

Our results from X-ray reflectivity and AFM clearly show the difference in reaction with water between defective oxide and defect-free oxide. In case of the defect-free oxide, the oxide layer does not react with water and 10 mM Pb contained solution while defect-rich oxide underwent significant changes in morphology and interfacial structure. It clearly shows that the defect free oxide has stronger passivity than defect-rich oxide.

Other possible mechanisms responsible for our findings include the role of polycrystalline and/or amorphous phases. The existence of amorphous phase may facilitate the chemical reaction between oxide layer and either water or Pb-contained solution. However, further investigation is needed to clarify the effects of these phases on the chemical reaction of nickel oxide layer.

## Conclusions

In summary, we investigated the role of defects in Ni oxide thin films on their chemical reaction in water and Pb contained solution. We used MBE grown NiO on MgO(001) substrate as the defect free NiO prototype, and NiO grown on Ni(110) single crystal as the one with defects. For the MBE grown NiO, we observed that the surface morphology changes in water and Pb-contained solution with monotonic increase in roughness. However, no significant changes were detected in the high-resolution X-ray reflectivity data. Meanwhile, in the case of native grown NiO on Ni(110), significant changes in both the morphology and atomistic structure at the interface were observed when immersed in water and Pb-contained solution. Furthermore, the reflected intensity decreased with exposure time, implying that the chemical reaction of oxide layer initiates from the defect sites and continues until the oxide undergoes a full phase transition into nickel hydroxide phase.

## Methods

### Materials

Two types of samples were used in this study. One is native grown nickel oxide on Ni(110) single crystal, and the other is epitaxially grown (001)NiO on MgO(001) substrate by molecular beam epitaxy (MBE) method. The reason we used NiO(110) for polycrystalline sample was based on our findings that it contains grain boundaries and mixed amorphous and crystalline phases that are ideal for studying the effect of defects on the reaction of NiO surface with Pb-contained solution^29^. And the reason we chose NiO(001) as our model system was because the epitaxially grown NiO(001) is the most studied system as well as the easiest to achieve epitaxial state close to single crystal.

Ni(110) single crystal (99.99%) with 10 mm diameter and 1.00 mm thickness was purchased from a commercial source (Princeton Scientific Corp.). The purchased sample surface was further mechanically polished with 0.03 μm alumina colloidal solution (pH ~3.5) followed by an electro-polishing with current density of 24.8 mA/mm^2^ and ~45 V for 50 s in mixture solution of 30% nitric acid – 70% methanol cooled by dry ice. After the electro-polishing, the sample was sputter-cleaned by Ar^+^ with 0.5 kV and 10 mA in 2.0 × 10^−5^ torr vacuum (10 mins) followed by annealing at 700 °C in 1.0 × 10^−8^ torr vacuum (5 mins) in an ultra-high vacuum (UHV) chamber at 33-ID-E beamline of Advanced Photon source (APS), Argonne National Laboratory. The sputtering and annealing procedure was repeated several times until an *in-situ* Reflection High-Energy Electron Diffraction (RHEED) pattern confirmed a homogenous single crystal surface. The cleaned surface was then exposed to high-purity oxygen gas in 4.0 × 10^−6^ torr for 4 mins to grow a thin native nickel-oxide layer. The sample was kept in a sealed container with minimal contact with air to avoid continuous growth of uncontrolled oxide layers with time until the X-ray reflectivity measurement was performed in helium environment first and in water in sequence. The average lattice constant of NiO polycrystalline film was 4.184 Å, which was measured from the cross-section TEM images. The strain states were complex, but the total axial strain of +3.2% corresponds to ~7 GPa of tensile stress in the surface normal direction and the total transverse strain was calculated to be −1.0%. The film thickness was 2.0~2.5 nm^29^

For a prototype of defect free nickel oxide, Ni(001) film was epitaxially grown on MgO(001) substrate (10 mm × 10 mm × 1 mm, one-side polished) by MBE. We estimate the out-of-plane and in-plane lattice constants to be 4.158Å and 4.213 Å^43^. The substrate was outgassed in UHV chamber up to 400 °C, and the nickel oxide was grown at 300 °C in 2.5 × 10^−6^ Ozone environments up to 7.2 nm, which is the NiO(001) film thickness. The film was characterized by atomic force microscopy (AFM) and the rms roughness was 0.718 nm.

### High resolution X-ray reflectivity

The native NiO layer grown on Ni(110) single crystal substrate was measured in helium environment and in bulk water using a thin-film cell, respectively, at room temperature. High resolution specular X-ray reflectivity measurements were performed at beamline 5-IDC of APS. The HRXR measurements basically scan the (00 L) crystal truncation rod (CTR) intensities along the surface normal direction, where *L* is the reciprocal lattice unit. The incident X-ray energy was 17.463 keV with ~8.0 × 10^10^ photons/sec beam flux. The reflected X-ray intensities were measured by Pilatus 100 K pixel array detector after 5 mm vertical and 5 mm horizontal slits. The background subtraction and the peak intensity integration were performed over ranges from *L* = 0.1 to 1.2 and *d*_(220)_ = 1.246 Å. As *qd* = 2π*L* relation, *L* = 1.2 corresponds to *q*_*max*_ = 6.0 Å^−1^, which allows the real space features to be resolved with ~0.5 Å resolution.

For the MBE grown nickel oxide, we moved beamline from 5ID-C to 33BM-C due to the beam schedule. The range of L is from 0.1 to 2.2 and *d*_(002)_ = 2.072 Å. Thus, *L* = 2.2 corresponds to *q*_*max*_ = 6.7 Å^−1^. The incident beam energy was 17.864 keV with ~6.0 × 10^8^ photons/sec. High-resolution x-ray reflectivity was measured in helium, water, and 10 mM Pb-solution with varying pH conditions (3, 7 and 11).

### *In-situ* atomic force microscopy (AFM)

In order to investigate the surface morphology changes in air, water, and Pb-contained solution, the film surfaces were characterized by *in-situ* AFM using ac mode at Oak Ridge National Laboratory. The scan size was 1 μm × 1 μm and the scan rate was 0.8~1.2 Hz. All images were taken using ultra-high frequency AFM probes with arrow shaped tip at the very end of cantilever coated by reflex aluminum.

## Additional Information

**How to cite this article**: Kim, J. *et al*. Effect of defects on reaction of NiO surface with Pb-contained solution. *Sci. Rep.* **7**, 44805; doi: 10.1038/srep44805 (2017).

**Publisher's note:** Springer Nature remains neutral with regard to jurisdictional claims in published maps and institutional affiliations.
